# Management of irreducible unilateral facet joint dislocations in subaxial cervical spine: two case reports and a review of the literature

**DOI:** 10.1186/s13256-018-1609-z

**Published:** 2018-03-21

**Authors:** Yu Zhou, Zhenyu Zhou, Lifeng Liu, Xuecheng Cao

**Affiliations:** 1 Department of Orthopedics, General Hospital of Jinan Military Command, Jinan, 250031 China; 2 Present address: Department of Orthopedics, Civil Aviation Hospital of Shanghai, Shanghai, 200025 China

**Keywords:** Cervical spine, Facet dislocation, Trauma, Surgery, Radiography

## Abstract

**Background:**

Skeletal and soft tissue damage are often associated with unilateral facet dislocations, which undoubtedly lead to instability of the spine and further increase difficulties in cervical reduction. This type of irreducible facet dislocation is usually accompanied with potential catastrophic consequences including neurological deficit and severe disability. Therefore, a consistent and evidence-based treatment plan is imperative.

**Case presentation:**

The literature regarding the management of traumatic unilateral locked cervical facet dislocations was reviewed. Two patient cases (a 30-year-old Asian man and a 25-year-old Asian woman) who suffered irreducible cervical facet dislocations were presented. These two patients received surgical treatments including posterior reduction by poking facet joints, adjacent spinous process fixation by wire rope banding, anterior plate fixation, and intervertebral fusion after the failure of skull traction and closed reduction. At the postoperative 24-month follow-up, intervertebral fusion was achieved and our patients’ neurological status improved based on the American Spinal Injury Association scale, compared with their preoperative status.

**Conclusions:**

Unilateral facet joint dislocations of subaxial cervical spine are difficult to reduce when complicated with posterior facet fractures or ligamentous injury. Magnetic resonance imaging can be beneficial for identifying ventral and dorsal compressive lesions, as well as ligamentous or capsule rupture. The combination of posterior reduction and anterior fixation with fusion has advantages in terms of clinical safety, ease of operation, and less iatrogenic damage.

## Background

Traumatic unilateral locked cervical facet dislocations are not rare injuries. Beyer *et al*. reported that approximately 12 to 16% of all cervical spine injuries are unilateral facet joint dislocations [1]. Skeletal and soft tissue damage are often associated with these dislocations including posterior facet fractures, ligamentous avulsion, and fibrous annulus rupture, which undoubtedly lead to instability of the spine and increase difficulty in cervical reduction [2]. Although some unilateral facet dislocations show relative stability in the dislocated position, significant hypermobility may occur when the dislocation is reduced because posterior ligamentous structures ipsilateral to the violated facet complex must be disrupted. Since this type of irreducible facet dislocation is often coupled with potential catastrophic consequences including neurological deficit and severe disability, it requires a consistent and evidence-based treatment plan [3]. Both sufficient radiological evaluation and appropriate surgical treatment are critical factors of increasing the cure rate. Based on the various injury characteristics of cervical spine trauma, the surgical method should be individualized for specific injuries. It has been acknowledged as a treatment principle that cervical stability is a major requisite in avoiding neurological deterioration [4, 5]. Following this absolute principle, a series of treatment procedures were carried out including posterior reduction by poking facet joints, adjacent spinous process fixation by wire rope banding, and anterior plate fixation for those irreducible unilateral facet dislocations. And, in this article, we reviewed the literature and summarized the clinical experiences in the management of irreducible locked unilateral facet joint dislocations of subaxial cervical spine, including diagnostic imaging and surgical technique.

## Case presentation

### Case 1

**Fig. 1:**
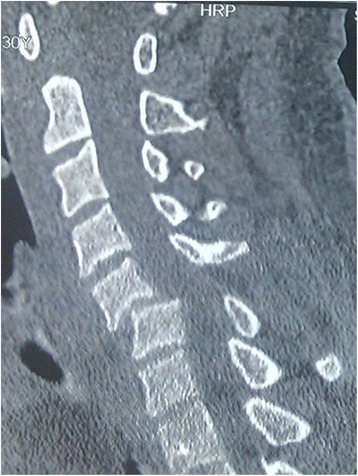
Computed tomography scan before treatment: approximately 50% anterior spondylolisthesis of C5 on the C6 body

**Fig. 2:**
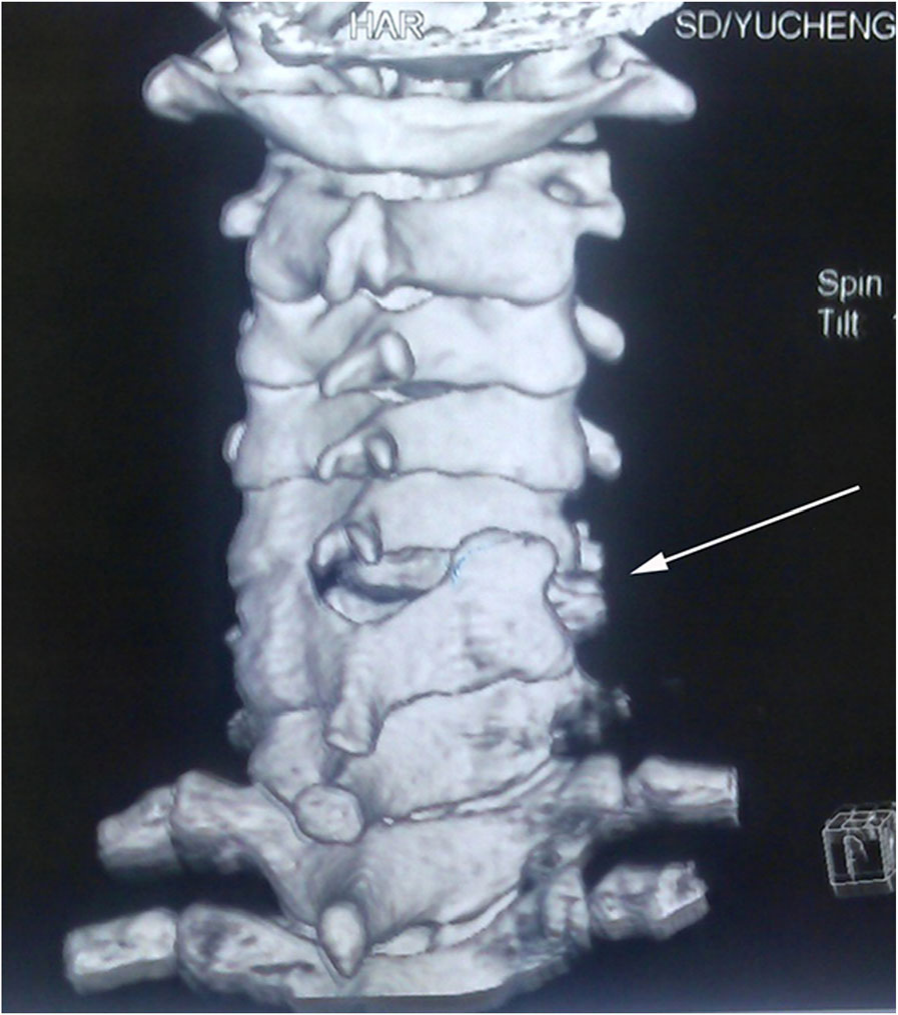
Three-dimensional computed tomography scan before treatment: locked facets of C5/6 (arrow)

**Fig. 3:**
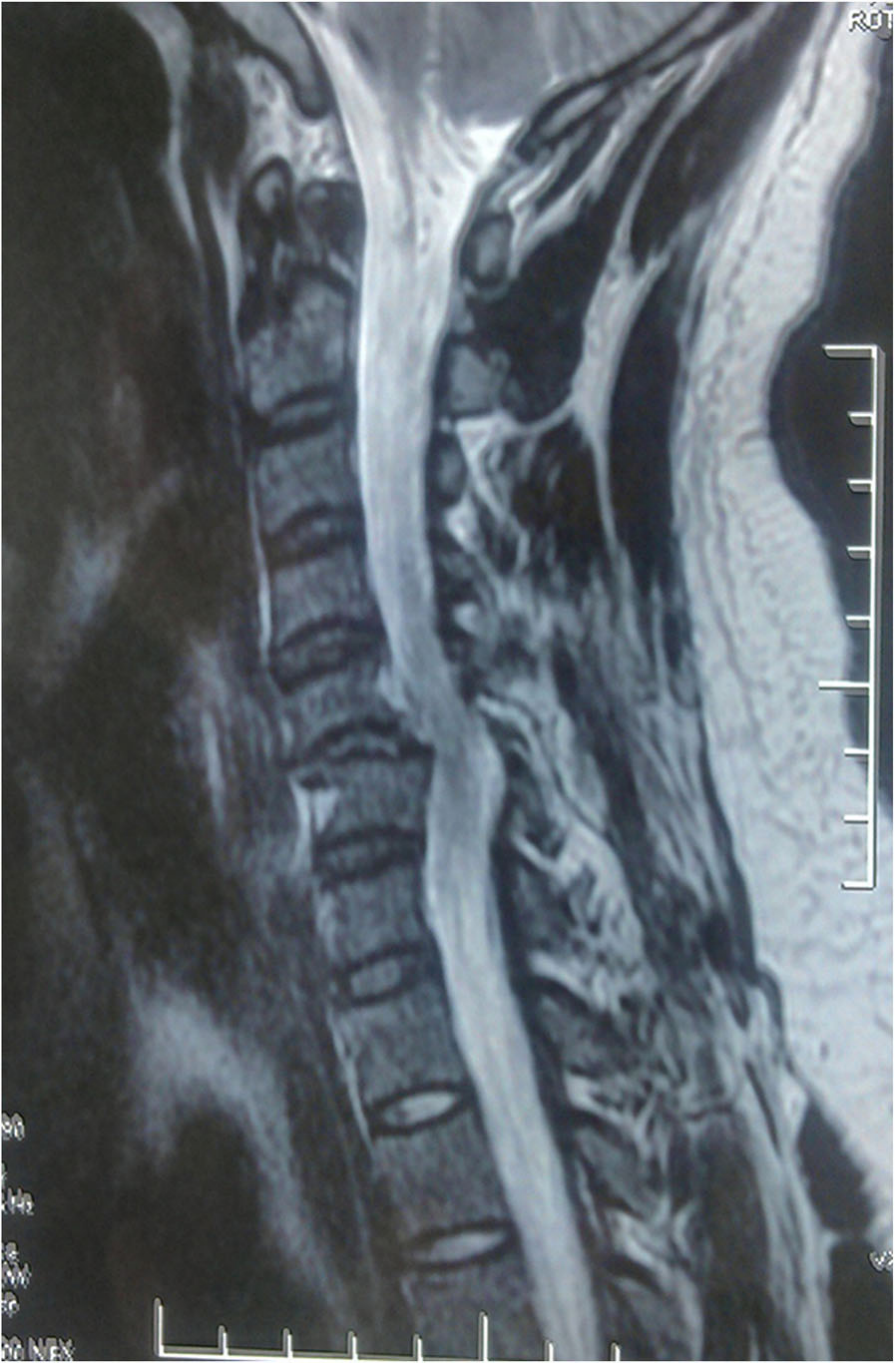
Preoperative magnetic resonance imaging: C5/6 disk rupture and extrusion and severe cord compression with increased signal changes

**Fig. 4:**
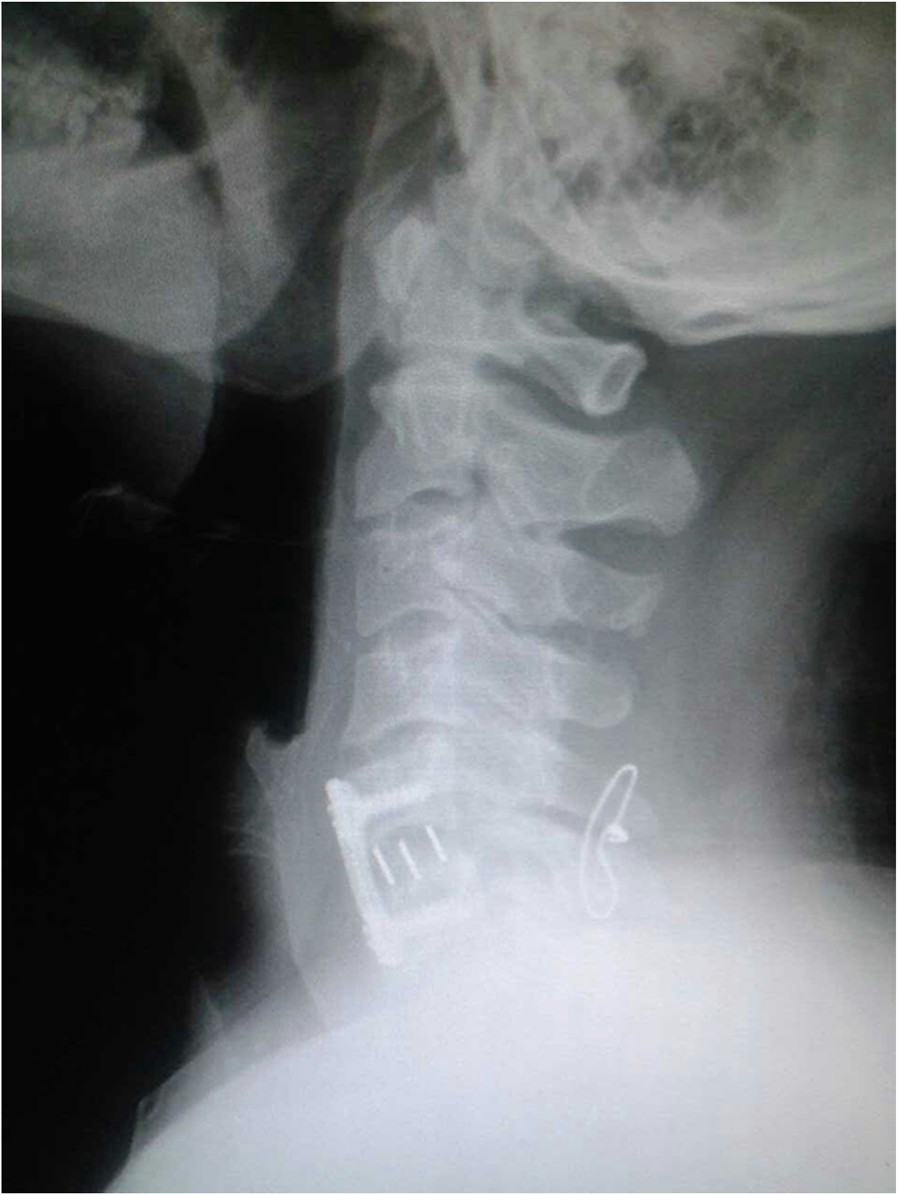
Postoperative C-spine anteroposterior radiograph: anterior fusion and posterior fixation with wire

**Fig. 5:**
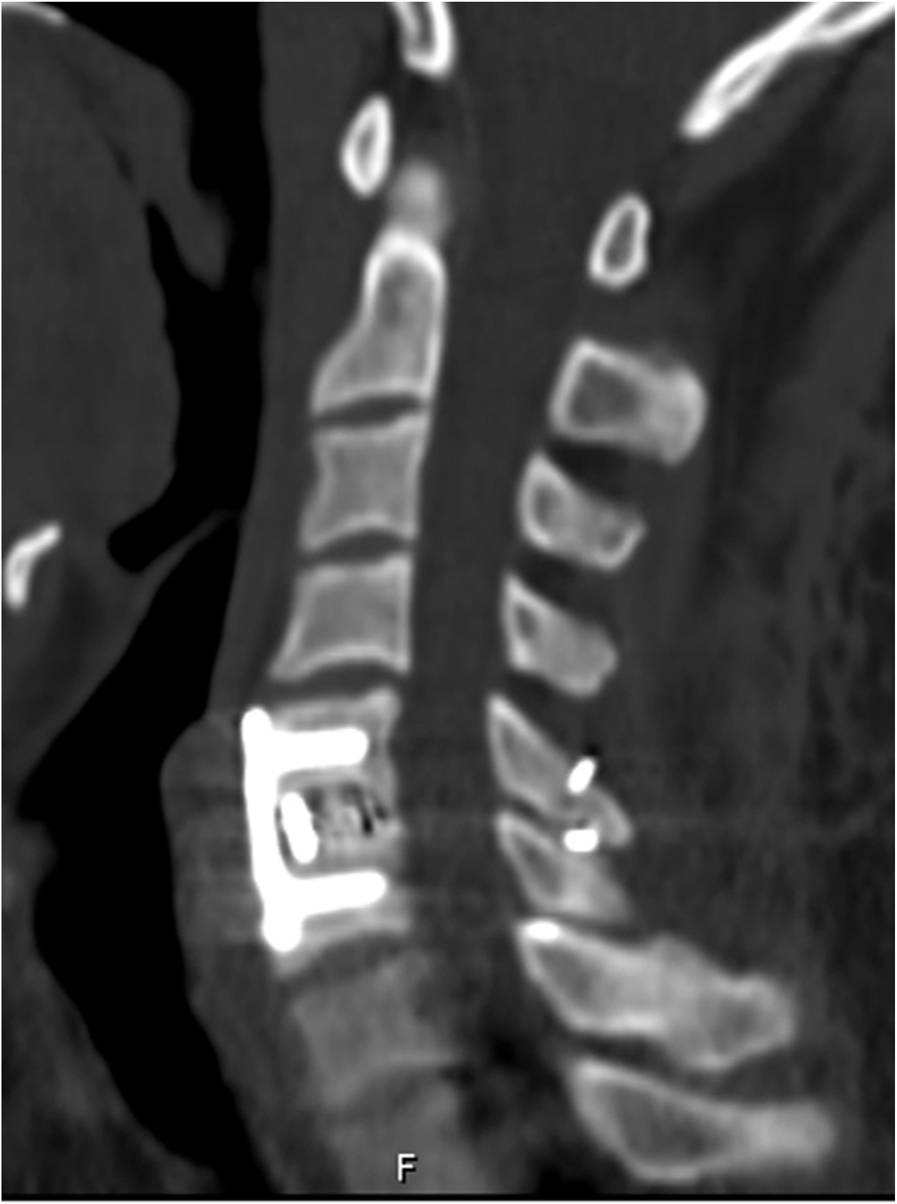
Computed tomography scan at the 24-month follow-up: excellent bony consolidation

A 30-year-old Asian man became paraplegic after being involved in a motor vehicle accident. An emergency computed tomography (CT) examination showed approximately 50% anterior spondylolisthesis of C5 on the C6 body (Fig. 1). A three-dimensional reconstruction revealed unilateral facet dislocations on the left side (Fig. 2). A neurological examination showed weakness of his bilateral biceps muscles (strength of 2 on a 5-point scale), impaired wrist extension, and numb skin of forearm. Immediate skull traction was performed to restore cervical alignment. Preoperative magnetic resonance (MR) imaging showed C5/6 disk rupture and extrusion and metamorphic signal intensity in his spinal cord and posterior ligament complex (Fig. 3). Cervical reduction from posterior approach was performed assisted with removal of a small part of C6 superior facet. The spinous processes of C5/6 were then stabilized with wire rope. Finally, anterior decompression and fixation of C5/6 were accomplished (Fig. 4). At the 12-month follow-up, sensory and motor function of extremities recovered compared to the preoperative status. A CT examination at 24 months postoperatively showed excellent bone consolidation and plate and screw stabilization (Fig. 5).

### Case 2

**Fig. 6:**
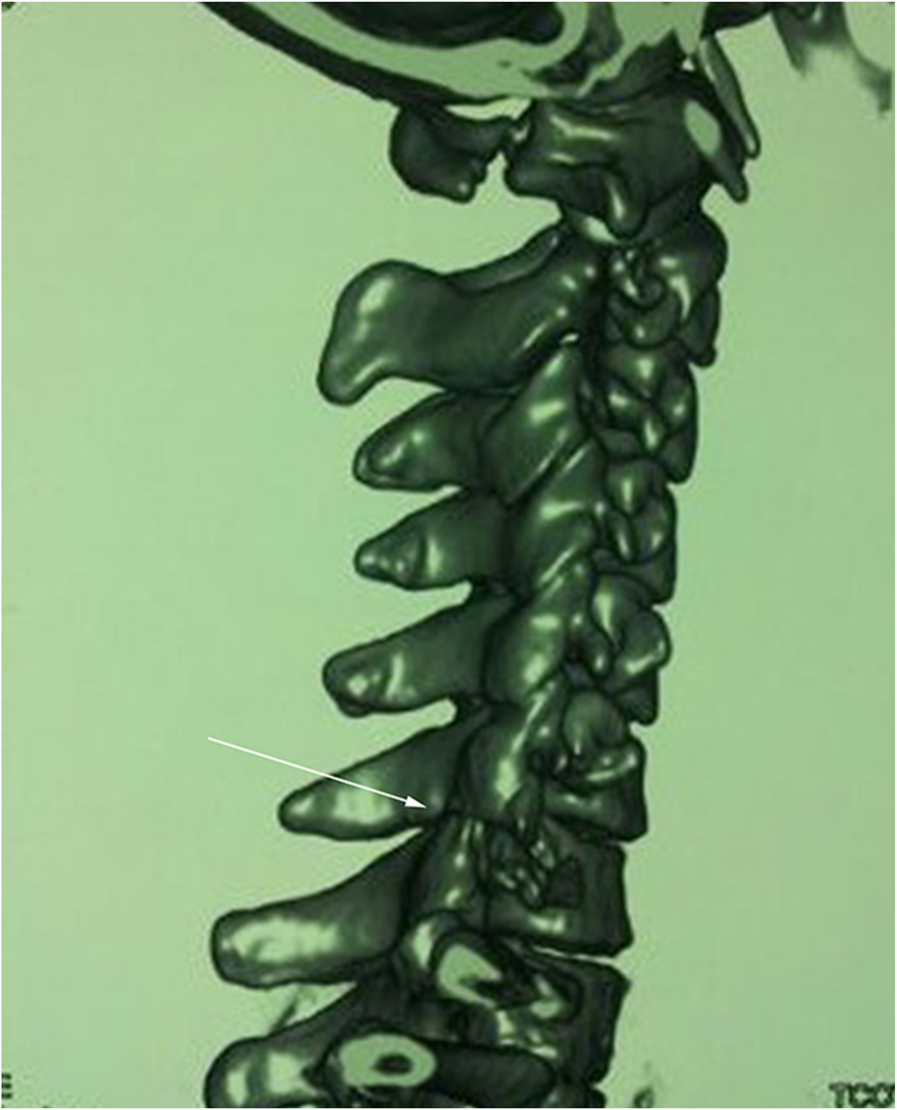
Three-dimensional computed tomography scan indicates articular process fracture and locked facets of C6/7 (arrow)

**Fig. 7:**
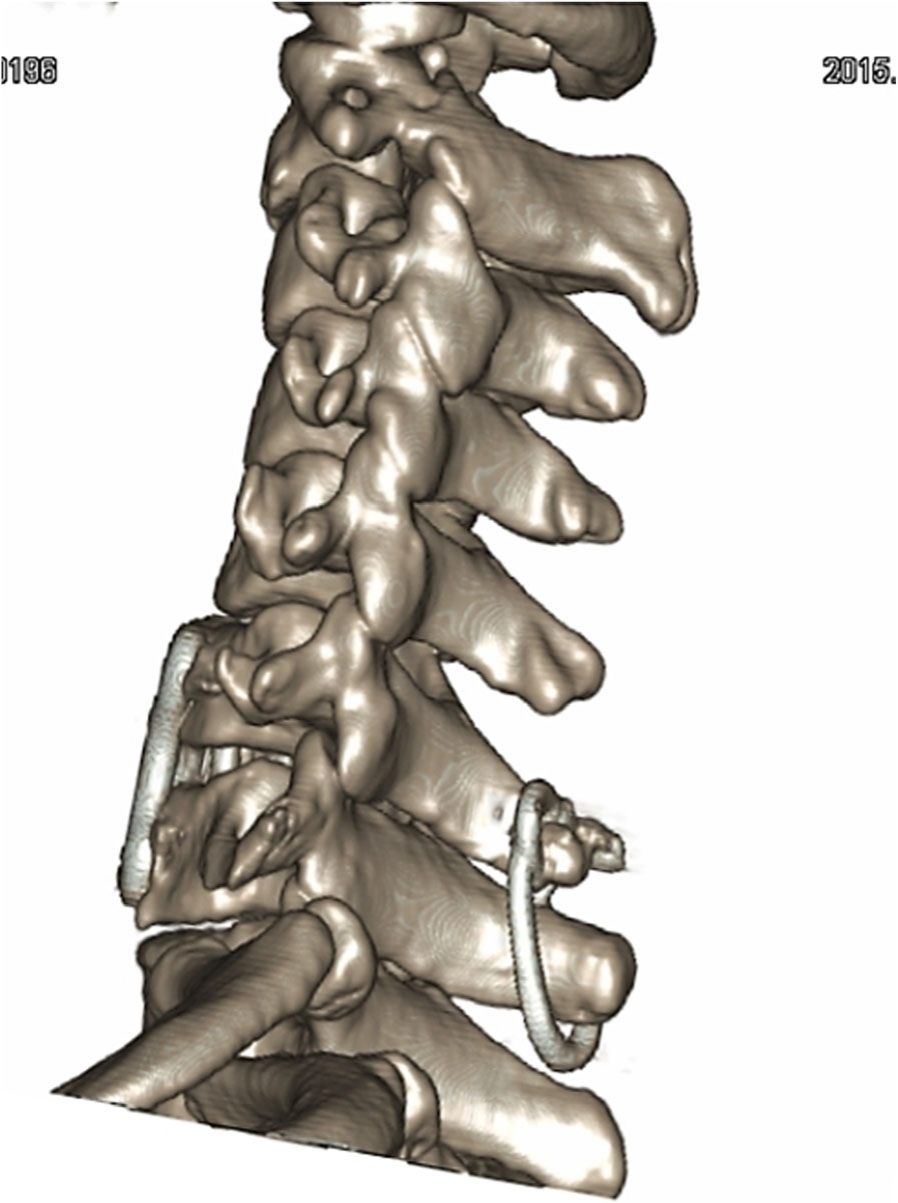
No loosening of the fixators was present at the 3-month follow-up

A 25-year-old Asian woman fell from a height of approximately 3 m, and her face contacted the floor first. On presentation to our hospital, she complained of severe neck pain, skin numbness of forearms, and weak limbs. A neurological examination showed impaired triceps brachii muscles (strength of 3+ on a 5-point scale). CT with three-dimensional reconstruction revealed an articular process fracture and unilateral facet dislocation on the right side at C6/7 (Fig. 6). MR imaging showed mild cord compression. Preoperative use of a cervical collar maintained her spinal alignment. Posterior reduction with wiring of the C6/7 spinous processes was performed after removal of the facet fragments. Anterior decompression and fixation using a cage with iliac bone and a plate was then performed. A halo vest was used postoperatively for the instability of the upper cervical spine due to atlas fracture. At 3 months postoperatively, loosening of the fixators had not occurred (Fig. 7), the aberrant skin sensation abated, and her muscle strength had improved. At the 6-month follow-up, consolidation of the graft bone was observed and her sensory and motor function had recovered completely.

### Surgical methods

The two patients were maintained in a supine position with a rigid cervical collar on admission, to prevent aggravation of neurological damage caused by an unstable spine and involuntary neck mobilization. Motor and sensory examinations were performed on the patients, following high-quality CT imaging and MR imaging. Early closed reduction with skull traction was applied prior to surgery, but failed.

After induction of general anesthesia, each patient was carefully turned to the prone position with the skull fixed to a head-holder device. Based on the exact orientation of the injured facet joint under intraoperative X-ray, an approximate 7-cm midline posterior incision was created. For unilateral facet dislocations, the interlocking zygapophyseal joint was identified, and a small periosteal elevator was inserted into the facet joint up to the front of inferior articular process of the dislocated vertebra. Through setting superior articular process of the lower vertebra as a supporting point, the inferior articular process of the upper vertebra was carefully pried backward to approach rear area of the lower articular process. The assistant surgeon simultaneously added gentle manual traction and slight rotation to the head. If these methods did not accomplish vertebral reduction, a rongeur was used to remove the zygapophyseal apex of the lower vertebra to assist reduction process. Reduction was confirmed by direct visualization. On affirmation of the cervical position, a steel wire rope was employed to truss up the spinous processes of the upper and lower cervical vertebrae.

Each patient was carefully turned to the supine position, and their neck was sterilized again. A median right cervical incision was made, and the soft tissue was separated deep within the space between the carotid sheath and trachea. The disk was removed by nucleus pulposus forceps, and thorough decompression was accomplished. Then a suitable polyetheretherketone (PEEK) cage or autologous bone was inserted into the intervertebral space. An anterior cervical plate was applied to secure the cage and obtain rigid anterior fixation.

## Discussion

Unilateral facet dislocations are more difficult to reduce than bilateral facet dislocations when complicated with posterior facet fractures or ligamentous injury, and require higher weights to achieve closed reduction [5]. Although some unilateral facet dislocations show relative stability when in the dislocated position, significant hypermobility may occur when the dislocation is reduced. Rorabeck *et al.* concluded that unilateral facet dislocations should be considered unstable injuries [6]. Without reduction of facet dislocation, cervical radicular pain arising from facet disruption and neurological deficits may be exacerbated.

### Diagnostic imaging

It has been documented that CT screening with three-dimensional reconstructions has a higher sensitivity (99%) and specificity (100%) for the detection of cervical spine injury, compared with the low sensitivity (70%) of plain film radiographs [7]. Despite many advantages of CT imaging, some occult cervical ligamentous injuries are also reported to be not easily detected by CT imaging technique [8]. In unstable cervical injuries, MR imaging is helpful in assessing the status of the discoligamentous complex. However, controversies surrounding the application of MR evaluation before closed reduction have historically existed among clinicians and researchers. Those investigators who disagree with the application of MR worried about the delay of cervical reduction and poor long-term outcomes caused by waiting period for MR results [9]. In fact, with the development of imaging techniques and widespread presence of MR devices in trauma centers, the worry becomes unnecessary. Nowadays, patients can receive MR examination immediately after CT scan and clinicians can obtain MR imaging at any time by the nosocomial computer network. By contrast, investigators who agree with the application of MR evaluation argue that no delay has been proven to be associated with any deterioration in neurologic function [10]. Based on the updated guidelines for the management of acute cervical spine and spinal cord injuries [11], although MR imaging is not recommended as equally as a CT scan, MR is still a promising technology in evaluating spinal cord injuries in subaxial cervical spine dislocations [3]. It has been suggested by Kaiser *et al*. that MR might be useful in the evaluation of soft tissue injuries, disk herniation, and hematomas, especially in neurologically injured patients with negative CT scan [12]. For facet dislocations associated with significant anterior disk herniation, it has been demonstrated that without reliable MR imaging before closed reduction, further retropulsion of a traumatic herniated disk prolapsing into the spinal canal during reduction maneuver would cause worsening neurological function [13]. In addition, MR may be critical for determining surgical approach as it assists in identifying ventral and dorsal compressive lesions.

### Surgical treatment

Surgical treatment is indicated for patients with the following features: unilateral facet dislocations with any facet fractures, ligamentous injury, persistent cord compression, or neurological deficit; difficulty in closed reduction or unavailable cervical stability; more than 4 points based on the injury severity score (Subaxial Injury Classification System). Once surgical treatment is chosen, neurological decompression should be performed as soon as possible, when the patients’ clinical conditions permit.

Various surgical techniques for these injuries have been described, including the anterior approach alone with fusion and plating, the posterior approach alone with pedicle screws or wires, and a staged anterior-posterior-anterior approach. No consensus exists on the best surgical strategy [5, 14]. Each of these options mentioned above has its own merits and drawbacks.

Before the 1990s, posterior instrumentation was preferred by many surgeons because it was generally recognized that cervical dislocations were associated with damage to the posterior ligament complex or muscles [15]. Therefore, it is important to achieve a tension band effect against the mechanism of injury by segmental fixation at the rear with wire rope, or rod and pedicle screw [16]. Fixation from posterior approach has many advantages, including deformity reduction under direct visualization, decompression by removing the fractured bone fragments which compress the spinal cord or nerve root, and stabilization by reconstruction of the posterior ligament complex [17]. However, posterior treatment alone might complicate the procedure, when accompanied with concomitant disk herniation or ventral compression. A disadvantage of the posterior approach was suggested by Ye and colleagues that more cervical segments shall be fixed and fused than in the anterior approach [14]. In other words, the soft tissues are more broadly damaged.

With the advent of titanium locked plates and threaded cages, anterior fixation and fusion provides satisfactory results: without dislodgement of bone graft, significant late displacement, or kyphotic angulation [15]. As for the anterior approach, direct decompression of the neural elements is achieved by removing ventral compressive structures such as disk and bone fragments. Furthermore, since the anterior approach is minor surgical trauma, it rarely causes iatrogenic soft tissue injury. However, the anterior approach alone can be technically challenging when intraoperative reduction is required [18], particularly when locked facet joints are irreducible by traction; it would increase the risk of secondary spinal injury in patients with neurological deficits under forcible reduction. Kim *et al*. [19] biomechanically demonstrated that combined anterior cervical decompression and fusion followed by posterior screw and rod fixation produces the greatest overall biomechanical stability. However, this procedure leads to extensive soft tissue injury, long operation time, large estimated blood loss volume, and high technical complexity of the operation [14].

Although both anterior and posterior approaches were applied in this study, the treatment method differed from the above-described procedure; the innovative surgical technique applied by our team minimized the postoperative complications to the greatest extent possible. Firstly, the posterior procedure was performed, in order to reduce locked facet dislocations instead of to accomplish final and solid fixation. Reduction of cervical spine was achieved by prying the dislocated joints by using a microdissector, as previously described. If the cervical spine was still hard to reduce, the zygapophyseal apex of the lower vertebra of locked facet would be drilled off or removed, to restore normal alignment. However, this position cannot be maintained without pressure and fixation when severe ligamentous rupture exists. Therefore, wire rope fixation of the adjacent spinous process was used as a posterior tension band. Wire rope has an advantage of decreasing the risk of neurological or vascular injury [20]. An anterior plate provides immediate stability to the cervical spine, by acting like a buttress in flexion and a tension band during extension. Combined with the interspinous wire rope, which restricts excessive flexion of the involved segments, anterior fixation proved to be sufficient for both segmental stability and final fusion.

The staged procedure described by Bartels and Donk [21] and Hassan [22] in the treatment of delayed traumatic cervical dislocations involved traction, posterior laminectomy or facetectomy, repeated traction, and anterior plating and fusion. This sequence led to long hospital stays. Moreover, patients had to remain on bed rest during the entire course of traction. In contrast, our technique has many advantages in terms of decreased blood loss, less operative time, and a shorter hospital stay. The main advantage of our method is its ease of operation, safe reduction via posterior approach, and decreased risk of progressive neurologic injury.

## Conclusions

Unilateral facet joint dislocations of subaxial cervical spine are difficult to reduce, when complicated with posterior facet fractures or ligamentous injury. Surgical decision making can be based on severity scores and CT scans; however, for those irreducible dislocations, MR imaging is recommended to identify ventral and dorsal compressive lesions (disk herniation, hematoma, bone segments) as well as ligamentous or capsule rupture. The application of MR imaging is critically important for determination of surgical approach and beneficial for avoidance of clinical worsening. From posterior approach, cervical reduction can be improved safely by direct facet poking, and wire rope fixation of adjacent spinous process compensates for cervical stability because of disrupted ligaments dysfunction. From the anterior approach, early decompression can be achieved with direct observation of the anterior pathology. The anterior plate provides immediate stability of the construct, by acting like a buttress in flexion and a tension band during extension. Combined with the posterior interspinous wire, which restricts excessive flexion of the involved segments, anterior fixation and fusion proved to be sufficient for both segmental stability and final fusion. This surgical method has advantages of safety, ease of operation, and less iatrogenic damage.
